# Clinical characteristics and prevalence of dihydropteroate synthase gene mutations in *Pneumocystis jirovecii*-infected AIDS patients from low endemic areas of China

**DOI:** 10.1371/journal.pone.0238184

**Published:** 2020-09-10

**Authors:** Mingli Zhu, Ning Ye, Jiru Xu

**Affiliations:** 1 Department of Microbiology and Immunology, School of Medicine, Xi’an Jiaotong University, Xi’an, Shaanxi, China; 2 Department of Clinical Laboratory, Hangzhou Xixi Hospital Affiliated to Zhejiang Chinese Medical University, Hangzhou, Zhejiang, China; 3 Department of Clinical Laboratory, Zhejiang Hospital, Hangzhou, Zhejiang, China; Universidad de Sevilla, SPAIN

## Abstract

Pneumocystis pneumonia (PCP) is an opportunistic and potentially life-threatening infection of AIDS patients caused by the fungus *Pneumocystis jirovecii* (*P*. *jirovecii*). Trimethoprim-sulfamethoxazole (TMP-SMX) is the most commonly used drug combination in the treatment and prophylaxis of PCP. However, with long-term use of this combination, mutations in the dihydropteroate synthase (DHPS) gene of *P*. *jirovecii* bring about the development of resistance. Data on the prevalence of *P*. *jirovecii* and its DHPS mutants in China, especially in low endemic areas, are still limited. Thus, in the present study, we measured the *P*. *jirovecii* infection rate among HIV-positive and AIDS (HIV/AIDS) patients with suspected PCP and investigated the relationship between CD4^+^ T cell count and PCP occurrence. As well as the polymerase chain reaction (PCR) analysis and sequencing, the restriction fragment length polymorphism (RFLP) method was used to analyze DHPS point mutation in *P*. *jirovecii* strains. *P*. *jirovecii* was detected in 40.82% of cases. The clinical symptoms and signs of PCP were not typical; with decreasing CD4^+^ T cell counts, PCP infection in HIV/AIDS patients increased. In only one case (1.67%), the patients’ DHPS gene could not be cut by the Acc I restriction enzyme. Furthermore, mutation at codon 171 was detected in 11 cases and no mutation was found at codon 57. Patients treated with sulfamethoxazole combined with Voriconazole or Caspofungin exhibited favorable results. After treatment, the symptoms of dyspnea were alleviated, and chest computed tomography findings showed the improvement of lung shadows. These indicated that the prevalence of DHPS mutations in *P*. *jirovecii* isolates in AIDS-PCP patients in the region was low. Thus, the contribution of gene mutations to treatment failure requires further research.

## Introduction

*Pneumocystis jirovecii* (*P*. *jirovecii*) is an opportunistic fungal pathogen that causes pneumocystis pneumonia (PCP) in immunocompromised individuals, especially in patients with HIV-positive and AIDS (HIV/AIDS). Due to the close correlation between HIV/AIDS and PCP, with the continuing growth in the number of patients with HIV/AIDS, the number of patients with PCP is also increasing. The clinical symptoms and signs of PCP are not typical, yet its progress is fast[1]. Therefore, rapid and accurate diagnosis is important for the prognosis and treatment of AIDS-related PCP.

Observation of *P*. *jirovecii* cysts and trophozoites by microscopy is currently the standard technique for the diagnosis of PCP[2, 3]. However, this method is time-consuming and exhibits low sensitivity, especially for lower-burden colonies[4, 5]. However, in recent years, the application of polymerase chain reaction (PCR) analysis has led to significant progress in the clinical diagnosis of PCP[1, 4].

Cotrimoxazole (sulfamethoxazole-trimethoprim) is the most common drug combination for prophylaxis and treatment of PCP. Cotrimoxazole targets two key enzymes in *P*. *jirovecii* folate synthesis: dihydropteroate synthase (DHPS) and dihydrofolate reductase (DHFR). The drug affects the synthesis of purine and pyrimidine, thus preventing the synthesis of nucleic acids and proteins, consequently killing *P*. *jirovecii*. However, with the wide use of sulfa drugs in the prevention and treatment of PCP, the relative resistance of *P*. *jirovecii* to them has gradually increased, seriously affecting the clinical effect of antibiotics on PCP patients[6, 7].

Numerous studies have indicated that sulfa prophylaxis failure and poor treatment outcomes are associated with DHPS gene mutations in the target organism[6–10]. Mutations associated with sulfa resistance have been observed in the DHPS gene at codons 55 and 57[11, 12]. However, data on the prevalence of *P*. *jirovecii* and its DHPS mutants in China, especially in low endemic areas, are still limited.

Unlike common bacteria that present visible colonies, *P*. *jirovecii* can not be cultivated *in vitro*. Thus, it is impossible to determine its drug resistance by traditional methods. Accordingly, PCR-based molecular methods to detect genes mutations in *P*. *jirovecii* are particularly important.

In the present study, we determined the prevalence of *P*. *jirovecii* in HIV-positive and AIDS patients with suspected PCP and the relative mutations of its DHPS genes in *P*. *jirovecii*-infected HIV/AIDS patients in a low endemic Chinese area and analyzed the clinical data of the patients retrospectively.

## Materials and methods

### Patients

This was a cross-sectional design study. A total of 147 HIV/AIDS patients with suspected PCP treated at the Department of Infection of Hangzhou Xixi Hospital from 2015 to 2016, presented pulmonary clinical symptoms and radiological features, were enrolled in this study. All patients were confirmed to be HIV-positive by chemiluminescence enzyme immunoassay (CLEIA) detection of anti-HIV-antibodies as well as P24 antigen (HISCL HIV Ag+Ab Assay Kit, Sysmex Corporation, Kobe, Japan) and Western blot analyses (HIV Blot 2.2, MP Diagnostic, MP Biomedicals Asia Pacific Pte., Ltd., Singapore). Demographic and other clinical information for these patients was obtained from medical records.

The diagnosis of PCP was based on clinical evidences and microbiological tests, such as microscopic examination or PCR. A patient with related clinical manifestations was diagnosed as PCP, only when there were microbiological evidences in the corresponding clinical samples, such as sputum, bronchoalveolar lavage fluid (BALF), or lung biopsy, as following conditions: (1) Fever, cough, chest tightness, progressive dyspnea, and respiratory distress in severe cases; (2) Chest X-ray and computed tomography (CT) examination evidencing diffuse reticular nodular interstitial infiltrate from the hilum of the lungs, or the appearance of ground glass-like shadows; (3) Hypoxemia, severe arterial oxygen pressure (PaO_2_), often less than 60 mmHg (1 mmHg = 0.133 kPa); (4) Blood lactate dehydrogenase (LDH) often above 500 mg/dL; (5) Diagnosis depends on etiological examination, such as *P*. *jirovecii* cysts/trophoblasts in sputum, bronchoalveolar lavage fluid (BALF), and/or lung biopsy as confirmed by microscopic examination[13]. Patients with positive PCP-DNA specimens who did not have typical clinical and/or radiological features for PCP were excluded from our analysis.

### Specimens

Samples (163; 16 BALF, 147 sputum) were obtained from 147 patients. Sputum was collected after oral cleaning, and 3% hypertonic normal saline after ultrasonic atomization was used to induce sputum drainage in patients with difficulty in sputum retention. Aseptic normal saline was injected into the lungs through an electronic fiber bronchoscope, and 10–20 mL of lavage fluid was retained after repeated irrigation.

The sputum samples were treated with *N*-acetyl-*L*-cysteine (NALC) solution, incubated at 37°C, and centrifuged at 3,000 rpm for 30 min, while the BALF was centrifuged at 3,000 rpm for 10 min. The supernatant was discarded and the precipitate was mixed with PBS. Direct examination (DE) was systematically performed on BALF and on sputum specimens (after cytospin procedure) by using and Gomori-Grocott staining. After processing, the samples were stored at -20°C for DNA extraction. Furthermore, we retrospectively collected clinical data on patient demographics, CD4^+^ T cell counts, treatments, and clinical outcomes.

The protocol for this research was approved by the Ethical Committee of Hangzhou Xixi Hospital, informed consent was obtained from all the study participants. All procedures and methods performed in this study were in accordance with the relevant international guidelines and regulations.

### DNA extraction

DNA was extracted from sputum and BALF specimens using a commercial kit (Biospin Fungus Genomic DNA Extraction Kit, Bioer, Japan) according to the manufacturer’s instructions. After washing the spin column, 100 μL DNA was collected in a 1.5 ml tube by centrifugation. Extracted DNA was then directly used for PCR or stored at -20°C for later experiments.

### DHPS gene amplification

The reported DHPS gene base sequences were obtained from Genebank, and the primer sequences were designed by ourselves and sent to Shanghai Yingweichiji Co., Ltd. to synthesize the primers for DHPS genes. For the DHPS gene, each sample amplification was carried out using DHPS-F [5’-TCTGAATTTTATAAAGCGCCTACAC-3’] and DHPS-R [5’- ATTTCATAAACATCATGAACCCG-3’] primers by PCR using Ex-Taq DNA polymerase (Ex Taq™ Version 2.0 plus dye, Takara, Japan) under the conditions described as follows: DNA was amplified by PCR in a PCR instrument (CFX-96,Bio-Rad). The PCR mixture contained template DNA, oligonucleotide primers (5 μM each), dNTPs (0.4 mM each), MgCl_2_ (4 mM), Ex-TaqDNA polymerase (1.25 U), and ddH_2_O to a total volume of 25 μL.

The PCR conditions were as follows: initial denaturation at 94°C for 5 min followed by 34 cycles of 94°C for 30 s, 60°C for 30 s, and 72°C for 1 min, followed by a final extension at 72°C for 10 min. To monitor for possible contamination, a negative control (ultrapure distilled water) was included in each PCR step.

The DNA marker and PCR products were placed into the well in the one percent of agarose gel, and 100 V electrophoresis was performed for 45 min. A gel imaging system was used to observe and photograph the target gene bands and those of the DNA marker.

### Purification and sequencing of PCR-amplified products

The DHPS amplified products were purified with a PCR purification kit (Axygen, NY, USA) according to the manufacturer’s instruction. Briefly, the target DNA in the agarose gel was cut under an ultraviolet lamp and placed in a 1.5 mL-tube. The gel weight was calculated according to the relationship of 100 mg = 100 μL. Three volumes of buffer was added, and the resultant solution was mixed and heated in a water bath at 75°C until the gel blocks melted completely. Different buffer solutions were added and mixed, the mixtures were centrifuged at each step, and the filtrate was discarded. The preparation tube was then put back into a 2-mL centrifuge tube and centrifuged at 12,000 ×g for 1 min. H_2_O (25 μL) was added to the center of the membrane. The tube was allowed to stand at room temperature for 5 min. The DNA was eluted by centrifuging at 12,000 ×g for 1 min. Finally, 1.5 mL of the DNA-containing liquid was stored at -20°C.

The purified products were sequenced by Shanghai Yingweijie group Co., Ltd. DHPS sequencing results were analyzed by Seqman software version (7.1.0(44.1)), and then compared with wild-type isolates (U66282).

### PCR-RFLP

Samples of the *P*. *jirovecii* from all positive cases were subjected to PCR-RFLP analysis to determine mutations at codons 55 and 57 of the *P*. *jirovecii* DHPS gene. In brief, PCR products were purified using a PCR Purification Kit (Axygen, NY, USA**)** according to the manufacturer's instructions. The purified products were digested with AccI or HaeIII restriction enzymes for 4 h at 37°C. Mutations were classified according to the pattern of band polymorphisms visualized on 1% agarose gels (Biowest Co., Ltd., Spain) stained with Goldview (Beijing Solarbio Science & Technology Co., Ltd., Beijing, China). The fragments of about 660 bp and 150 bp positions were expected.

DHPS Acc I and Hae III single enzyme digestion reaction system was as follows: the amount of DNA required was <1000 ng, which was mixed with 10× Buffer (2 μL) and Acc I or Hae III restriction enzyme (1 μL). Finally, H_2_O was added to make the volume up to 20 μL, and the system was placed in a 37°C water bath for 4 h. After enzyme digestion completed, the reaction was terminated by adding 6× Loading Buffer, and the enzyme digestion product was subjected to 100 V electrophoresis using 1% agarose gel for 45 min. After electrophoresis, the gel imaging system was used to observe and take photos. The target gene bands were determined according to the DNA standard.

### T lymphocyte counts

Fluorescent antibody and 100 μl EDTA-K2 anticoagulation peripheral blood were placed in a plastic tube. After blending, they were placed at room temperature in the dark for 15 min and then hemolyzed and fixed using a TQ-PREP instrument. CD3^+^, CD4^+^, CD8^+^ T cells, NK cells, and CD19^+^ lymphocytes were measured by flow cytometry (FC-500, Beckman Coulter, USA).

### Treatment and prognosis of AIDS patients with PCP

Anti-PCP treatment with Cotrimoxazole (three tablets t.i.d.) was administered for ca. 3 weeks, and the dose and course of treatment were adjusted according to the condition of the patient. Voriconazole injection (300 mg intravenous drip twice on the first day, followed by 200 mg intravenous drip q.12 h) was also administered as an anti-fungal treatment. This treatment lasted for ca. 10 days, and 200 mg orally b.i.d. (or 70 mg intravenously on the first day, followed by 50 mg intravenously q.d.) was continued as appropriate. Timely administration of highly active antiretroviral therapy (HAART) was performed to achieve immune reconstruction and reduce opportunistic infections. For patients with excess sputum, coughs, and poor pulmonary ventilation, mucosolvan was administered to relieve cough and reduce sputum, while methylprednisolone reduced pulmonary inflammation and exudation and improved ventilation diffusion function. Other treatments included nutritional support, bed rest, oxygen, maintaining water and electrolyte balance, and treatment of other opportunistic infections.

### Statistical analysis

Continuous variables were expressed as the means and standard deviations or medians and ranges as appropriate. Categorical variables were summarized as the counts and percentages in each category. To compare the continuous variables for data of different patient groups, a two-tailed t-test or Mann-Whitney U test was used as appropriate. Chi-square tests or Fisher’s exact tests were used for categorical variables as appropriate. All statistical analysis was performed with SPSS software version 19.0. P-value (two-sided) less than 0.05 was considered statistically significant.

## Results

### Demographic characteristics

We considered a total of 147 patients diagnosed with HIV/AIDS according to accepted guidelines[13, 14]. Of these 147 patients with suspected PCP, 123 (83.67%) were male and 24 (16.33%) were female, and their mean age was 43.17 ± 11.96 years old (22 to 75 years old).

### DHPS gene amplification

**Fig 1:**
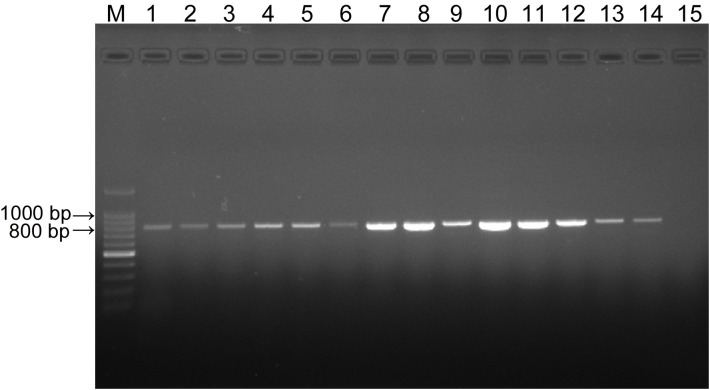
PCR amplification results for the DHPS gene. M: 100 bp DNA marker; Lanes 1–14: DHPS gene amplification product of PCP patients; Lane15: negative control.

The electrophoresis results of several representative DHPS genes with positive PCR amplification are shown in Fig 1. The positive samples show a single target band of ca. 810 bp, and no DNA bands are observed for the negative control. Consistent DHPS gene amplification results were seen in the samples between BALF and sputum taken in the same patients. According to the comprehensive DHPS PCR tests (60 out of 147 patients were positive), hexamine silver staining results (25 patients were positive for Gomori-Grocott staining), and clinical manifestations, 60 of the 147 patients (40.82%) were PCP positive, they were also classified as AIDS stage patients according to the guidelines[13, 14]. Among them, 49 cases of males (81.67%) and 11 cases of females (18.33%) were included, with ages (mean ± SD) of 43.02 ± 11.91 years old. There were 87 patients with HIV/AIDS without PCP, including 74 males (85.06%) and 13 females (14.94%), with age (mean ± SD) 43.28 ± 12.07 years old. There were no statistically significant differences in gender, age, and other basic data between the two groups (*P* >0.05).

### Clinical manifestation

Of the 60 AIDS cases complicated with PCP, most came to the hospital due to fever, cough, chest dyspnea, and acute symptoms, among which 48 cases had a fever (80.00%) with temperatures of 38–40°C. There were 32 cases (53.33%) with a dry cough, 9 cases with sparse cough, 10 cases with white phlegm, 1 case with white bubble phlegm, and 1 case with yellow bubble phlegm. Forty-six patients (76.67%) had urgent stuffy chest symptoms, 41 patients (68.33%) had fever and cough symptoms, and 34 patients (56.67%) had a fever, cough, and urgent chest symptoms simultaneously. At the same time, most patients also had other opportunistic infections, as shown in Table 1.

**Table 1:** Opportunistic infection rates of the 60 AIDS-PCP (%) patients considered in this study.

Opportunistic infections	Cases	Infection rate (%)
**Oral candidiasis**	25	41.67
**Bacterial pneumonia**	19	31.67
**Cytomegalovirus infection**	19	31.67
**Syphilis**	18	30.00
**Viral hepatitis B**	7	11.67
**Mycoplasma pneumonia**	6	10.00
**Pulmonary tuberculosis**	2	3.33
**Viral hepatitis C**	1	1.67
**Herpes simplex**	1	1.67
**Herpes zoster**	1	1.67
**Tubercular meningitis**	1	1.67
**Cryptococcus meningitis**	1	1.67
**Staphylococcal sepsis**	1	1.67
***Talaromyces marneffei***	1	1.67
**Only PCP**	13	21.67

PCP: pneumocystis pneumonia; AIDS-PCP: AIDS-related PCP

To be treated with Cotrimoxazole, AIDS patients also received HAART to promote immune reconstitution and reduce opportunistic infections. Of the 60 patients, only one died due to other various complications; the rest, whose chest CT results initially showed lung shadows, improved after treatment.

### Radiological examination

The radiological examination of 60 patients with AIDS-PCP mainly showed reduced brightness of permeability in both lungs, increased texture in both lungs, and patchy, floccular, strip, and ground-glass high-density fuzzy shadows in both lungs. See Table 2 for details.

**Table 2:** Radiological imaging characteristics of the 60 patients with AIDS-PCP.

Imaging features	Cases	Ratios (%)
**Bilateral brightness reduction**	23	38.33
**Increased veins in both lungs bilateral markings**	16	26.67
**Bilateral patche shadows**	25	41.67
**Flocculent shadows**	8	13.33
**Striped vague shadows**	6	10.00
**Ground-glass shadows**	43	71.67

PCP: pneumocystis pneumonia; AIDS-PCP: AIDS-related PCP

### Relationship between lymphocyte subsets and PCP

The relationship between CD4^+^ T cell level and AIDS-PCP is summarized in Table 3. The lymphocyte number, CD3^+^ T cell number, CD4^+^ T cell number, CD8^+^ T cell number, and CD4^+^/CD8^+^ T cell ratio of the AIDS-related PCP patients are significantly lower than those of HIV/AIDS without PCP (*P* <0.01). However, there are no statistically significant differences in NK cell count and total B cell count between the two groups (*P* >0.05), as shown in Table 3.

**Table 3:** Comparison of peripheral blood lymphocyte subsets in HIV/AIDS patients with and without PCP.

Groups	HIV/AIDS patients without PCP	AIDS-PCP patients	*P-*value
**Number of patients**	87	60	
**Lymphocytes (×10**^**6**^**/L)**	960(580, 1580)	715(510, 995)	0.004
**CD3**^**+**^ **T cells (×10**^**6**^**/L)**	739(420, 1164)	447(351, 723)	0.001
**CD4**^**+**^ **T cells (×10**^**6**^**/L)**	104(28, 204)	21(10, 58)	<0.001
**CD8**^**+**^ **T cells (×10**^**6**^**/L)**	598(315, 939)	384(294, 593)	0.008
**NK cells (×10**^**6**^**/L)**	88(54, 198)	88(46, 123)	0.170
**B cells (×10**^**6**^**/L)**	62(37, 117)	64(38, 117)	0.771
**CD4**^**+**^ **T/CD8**^**+**^ **T cell ratios**	0.15(0.06, 0.30)	0.04(0.02, 0.11)	<0.001

PCP: pneumocystis pneumonia; AIDS-PCP: AIDS-related PCP; NK cells: natural killer cells

Among the 87 cases of HIV/AIDS patients without PCP, the CD4^+^ T cell numbers of 42 cases (48.28%) are below 100 ×10^6^/L, while 45 patients (51.72%) have CD4^+^ T cell numbers >100 ×10^6^/L, among which 22 cases (25.29%) have >200 ×10^6^/L, with the highest value being 1,382 ×10^6^/L. Compared with the non-PCP group, the CD4^+^ T cell number in the PCP group is significantly decreased (*P* <0.01). Therein, the CD4^+^ T cell numbers of 51 patients (85.00%) are below 100 × 10^6^/L, those of 44 patients (73.33%) are below 50 ×10^6^/L, and only 9 patients (15.00%) have CD4^+^ T cell numbers >100 ×10^6^/L, but all are below 200 ×10^6^/L.

Among the 75 patients for whom the CD4^+^ T cell counts were below 50 ×10^6^/L, 44 patients (58.67%) were infected with *P*. *jirovecii*. Among the 18 patients for whom the CD4^+^ T cell counts were between 51× 10^6^/L and 100 ×10^6^/L, 7 patients (38.89%) were infected with *P*. *jirovecii*. Among the 32 patients for whom the CD4^+^ T cell counts were between 101 × 10^6^/L and 200 × 10^6^/L, 9 patients (28.13%) were infected with *P*. *jirovecii*. PCP was not found in the 22 patients with CD4^+^ T cell count >200 ×10^6^/L, as shown in Table 4.

**Table 4:** Different CD4^+^ T cell numbers associated with PCP infection in HIV/AIDS patients.

CD4^+^ T cells (×10^6^/L)	Cases	AIDS-PCP patients	Percentage (%)	*P-*value
**≤ 50**	75	44	58.67	0.130^a^
**51~100**	18	7	38.89	0.004^b^
**101~200**	32	9	28.13	0.434^c^
**>200**	22	0	0	

PCP: pneumocystis pneumonia; AIDS-PCP: AIDS-related PCP.

^a^Comparison between groups with CD4^+^ T cell number ≤50× 10^6^/L and group with 51 ×10^6^/L–100 ×10^6^/L.

^b^Comparison between groups with CD4^+^ T cell number ≤50 ×10^6^/L and group with 101 ×10^6^/L–200 ×10^6^/L.

^c^Comparison of CD4^+^ T cell number between the group with 51 ×10^6^/L–100 ×10^6^/L and group with 101 ×10^6^/L–200 ×10^6^/L.

### Sequencing and analysis of DHPS genes

**Fig 2:**
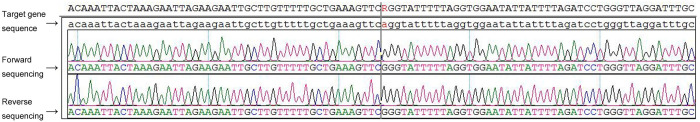
Sequence diagram of DHPS PCR amplification products.

Sequencing results for the DHPS gene were completely consistent with the target gene sequences in Genbank. The results of Gomori-Grocott staining and PCR were the same in 16 patients with both sputum and alveolar lavage. The DHPS gene of 147 HIV/AIDS patients was detected, and 60 cases were positive. The *P*. *jirovecii* from 11 patients exhibited synonymous mutations in DHPS gene codon 171, which was mutated from TCA to TCG, without affecting the amino acid sequence, as shown in Fig 2.

### RFLP analysis of PCR products of DHPS gene

**Fig 3:**
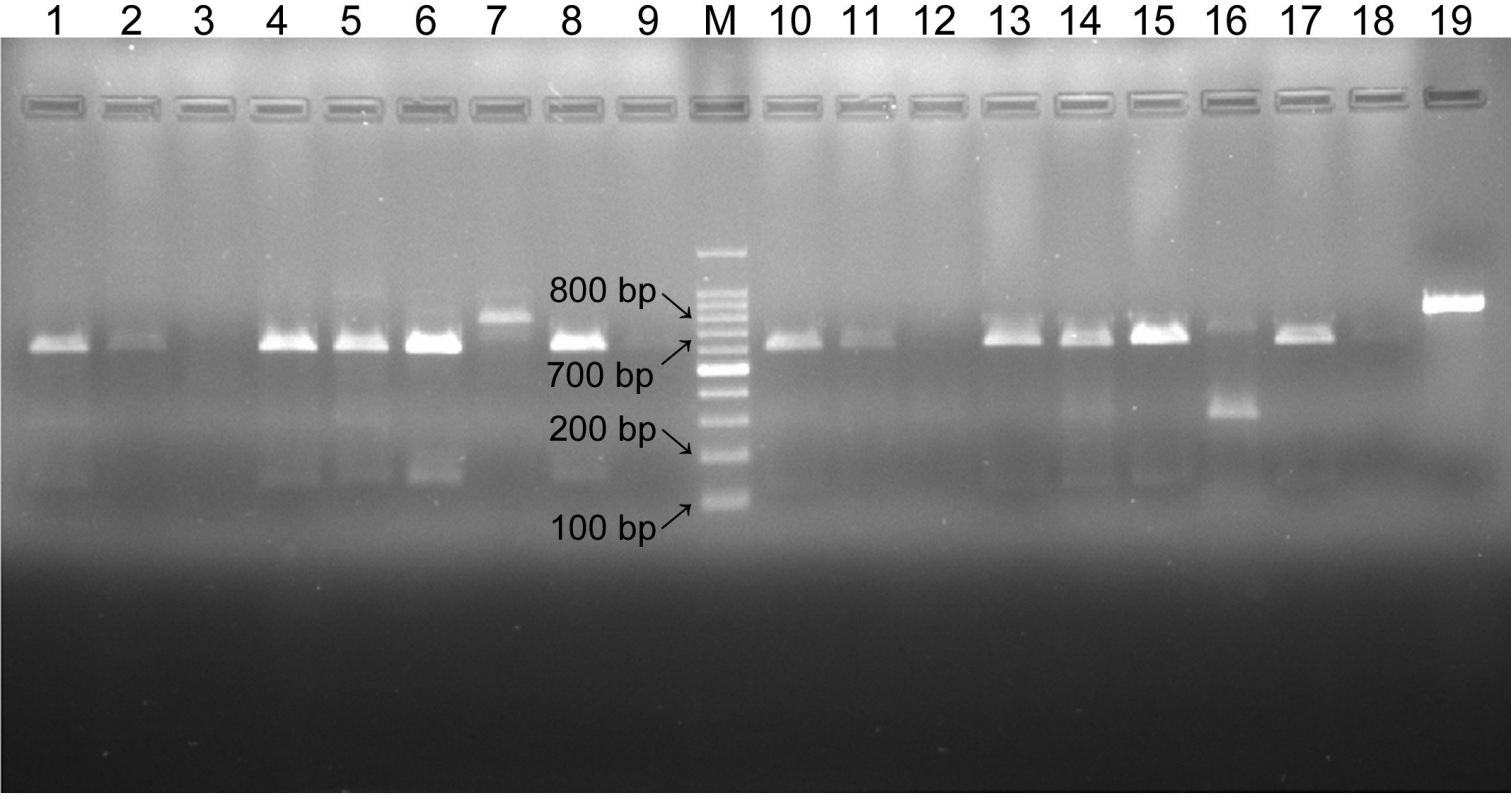
A typical result of Acc I and Hae III restriction enzyme analysis of DHPS gene PCR products. M: 100 bp DNA marker; Lanes 1–9: Results of Acc I enzyme digestion of DHPS gene amplification products; Lanes 10–18: Results of Hae III digestion of DHPS gene amplification products; Lane 19: Control (no enzyme).

Of the 147 patients, 60 tested positive for *P*. *jirovecii*. Samples of the *P*. *jirovecii* from all 60 positive cases were subjected to PCR-RFLP analysis to determine mutations at codons 55 and 57 of the *P*. *jirovecii* DHPS gene. DHPS gene PCR products were digested by restriction enzymes Acc I and Hae III. Bands around the 660 bp and 150 bp positions were expected. In the present study, in only 1 case (1.67%), the patients’ DHPS gene could not be cleaved by the Acc I enzyme, indicating 55th codon mutations. The other 59 cases (98.33%) of patients showed no DHPS gene mutation related to drug-resistant genes. A typical enzyme electrophoresis results are shown in Fig 3.

We also investigated the association between DHPS mutation and drug therapy. From clinical data, we found that 60 cases of AIDS-PCP had satisfactory treatment results for PCP. AIDS-PCP patients with showing *P*. *jirovecii* DHPS gene mutations in the 55th codon and the 171th codon were improved after treatment.

## Discussion

*P*. *jirovecii* is an opportunistic pathogen that causes PCP in immunocompromised and immunosuppressed patients[3, 15]. PCP is a major cause of morbidity and mortality in AIDS patients[16] and is considered an AIDS signal disease. Despite its decreasing incidence with the use of combined antiviral therapy, PCP is still a serious health concern for people living with HIV/AIDS[17]. In this study, clinical data analysis and mutation analysis of *P*. *jirovecii* DHPS genotypes was performed. Specific attention was paid to the possible correlation between Cotrimoxazole prophylaxis/therapy and gene mutation.

The major reasons for HIV/AIDS-PCP patients being admitted to the hospital are fever, cough, chest tightness, and shortness of breath. The overall positive rate in our single-center study is 40.82%, which is higher than those reported previously[18–20]. This may be attributed to the patient selection standard and the sensitivity of the methods used for specimen detection.

Previous studies have indicated that opportunistic infection in HIV/AIDS patients is strongly correlated with CD4^+^ T cell count[21]. When the CD4^+^ T cell count was less than 200 ×10^6^/L, the incidence of PCP infection increased, and with a further decrease of the CD4^+^ T cell level, the risk of complication with PCP infection was increased[22–24]. In the present study, the CD4^+^ T cell counts of the AIDS-PCP patients were less than 200 ×10^6^/L, and most were lower than 100 ×10^6^/L. The incidence of PCP infection in patients with CD4^+^ T cell counts lower than 100×10^6^/L was 54.83%, which is significantly higher than the group whose CD4^+^ T cell count was 101–200 ×10^6^/L (*P <*0.05). These results show that the incidence of PCP infection is CD4^+^ T cell-count-dependent and that the prevalence of PCP infection in HIV/AIDS patients increases with decreasing CD4^+^ T cell count.

Currently, Cotrimoxazole is the first-line treatment for PCP infection. With its widespread use for the prevention and treatment of PCP in HIV/AIDS patients, PCP morbidity and mortality has decreased significantly, but resistance to the drug has gradually increased[7]. At present, the incidence and mortality of HIV/AIDS-related PCP in China are increasing gradually, and there is little published larger sample size research on the relationship between *P*. *jirovecii* DHPS mutation and drug resistance in PCP patients.

The epidemic trend of HIV/AIDS in the Zhejiang province of China from 1985 to 2009 could be divided into three stages: From 1985 to 1996, 26 cases of HIV infection/AIDS were reported. From 1997 to 2003, 479 cases were reported. During the period of rapid growth from 2004 to 2009, 4614 cases of HIV infection/AIDS were reported[25]. The reported HIV infections have reached 11,357 by the end of 2012[26]. On October 31, 2017, there were 22,830 living HIV/AIDS patients in Zhejiang province. The HIV/AIDS epidemic in Zhejiang province is at a relatively low level, far less than that in Henan and Yunnan province[26]. At present, PCP infection in low AIDS endemic areas is rarely reported.

*P*. *jirovecii* cannot be cultured *in vitro*, making the detection of drug-resistant target gene mutations particularly difficult. Parobek *et al* detected the specific site of *P*. *jirovecii* DHPS mutations for drug resistance to sulfonamides and found that harbored mutations in DHPS are consistent with positive directional selection for sulfa resistance[6].

A number of studies have shown that DHPS gene mutations in *P*. *jirovecii* isolates are associated with resistance to sulfa drugs[7–10, 27]. Some studies reported that DHPS mutations would be expected to be more important than DHFR mutations to the development of potential TMP-SMZ drug resistance[28]. Numerous studies have focused on DHPS non-synonymous polymorphisms associated with sulfa drug resistance at codons 55 and 57, where amino acids 55 and 57 change from threonine to alanine and proline to serine, respectively[11, 12]. These mutations have an effect on the enzymatic structure and may confer sulfa resistance since these amino acids are located at the active site of the enzyme[29].

In the results of the present larger sample size study, only one case (1.67%) exhibited codon 55 mutation and the majority of cases showed the wild-type genotype. This result is consistent with those of previous studies of Chinese people. Kazanjian *et al* revealed a DHPS gene mutation rate of 6.7% (1/15) in AIDS-PCP in Beijing[30], Deng *et al* identified DHPS mutations in 12.0% (3/25) patients from Guangzhou[31], the prevalence of *P*. *jirovecii* DHPS mutations in this study is not significantly higher than those of these previous studies (*P* = 0.858, *P* = 0.137, respectively). In addition, Li and Wang *et al* did not find mutations in the DHPS gene in *P*. *jirovecii* in 10 and 18 HIV-infected patients in Guangzhou[32] and zhejiang[33], respectively. And no common DHPS gene mutations of *P*. *jirovecii* were detected in the HIV-negative PCP patients in Beijing[34]. The overall prevalence of *P*. *jirovecii* DHPS mutations in the study remains low in the region, which is also similar to that reported in other developing countries, such as India, Thailand, and Turkey[20, 35, 36], but the mutation rate is much lower than that in some western developed countries[30]. This may be related to the late development of sulfa drugs for prevention and treatment in developing countries and the increasing use of combination therapy. The prevalence of these genotypes varies depending on geographical location, it can be transmated from person-to-person[27]. Due to the low AIDS endemic areas, this transmissin might be also low. Our results for DHPS mutation suggest that the selective pressure of sulfa drugs in China has not reached the levels in developed countries. Furthermore, codon 171 showed the same synonymous mutation in 11 out of 60 samples, similar findings have been reported previously[20, 37, 38]. However, this mutation would have no effect on enzymatic activity and may not confer sulfa resistance.

In addition, we found that 60 AIDS-PCP patients were treated with Cotrimoxazole combined with Voriconazole or Caspofungin and that these treatments had a satisfactory effect. Among them, one case with *P*. *jirovecii* DHPS gene mutation in codon 55 improved after treatment, and no drug resistance or treatment failure occurred. Therefore, in order to reduce the impact of DNA mutation related to drug resistance of *P*. *jirovecii*, systematic measures should be conducted, including using antimicrobials strictly according to the indications.

## Conclusions

In conclusion, our study showed that atypical mutations at codons 55 and 57 in the *P*. *jirovecii* DHPS gene were low for AIDS-PCP patients in a low endemic area in China. However, with the increase of HIV/AIDS occurrence in China and the application of sulfonamides in its treatment, it may be speculated that ongoing use of this treatment strategy will lead to the occurrence of a higher rate of mutation in *P*. *jirovecii*. Thus, it is very important to continue monitoring the prevalence of sulfa-drug-resistance mutations in *P*. *jirovecii*.

## Supporting information

S1 Raw images Original gels of Figs 1–3. (DOCX)

S1 Data Raw data of 147 HIV/AIDS patients with suspected PCP. (XLSX)
