# Supplementary material for: Clinical characteristics and prevalence of dihydropteroate synthase gene mutations in Pneumocystis jirovecii-infected AIDS patients from low endemic areas of China
Source: PLoS One. 2020 Sep 10;15(9):e0238184. doi: 10.1371/journal.pone.0238184 (PMC7482917; doi:10.1371/journal.pone.0238184)
Supplement: S1 Raw images — (DOCX) [file pone.0238184.s001.docx]

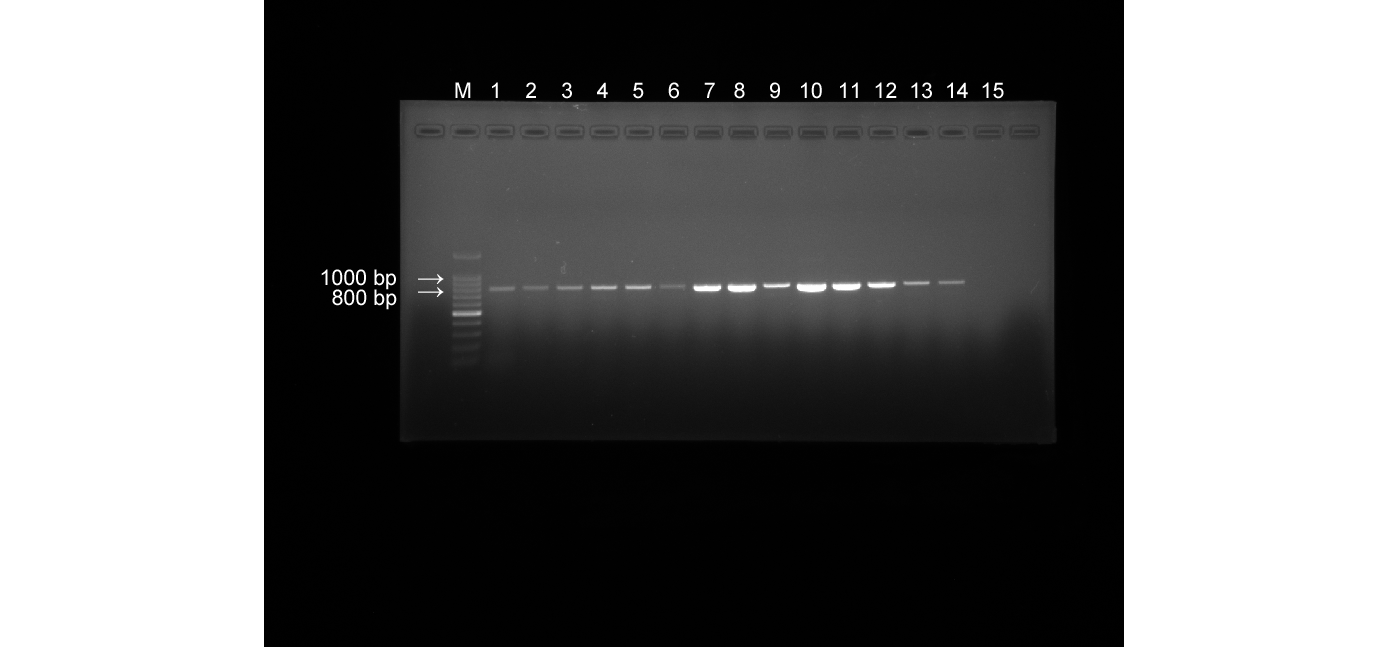


**Fig 1. PCR amplification results for the DHPS gene.** M: 100 bp DNA marker; Lanes 1–14: DHPS gene amplification product of PCP patients; Lane15: negative control.


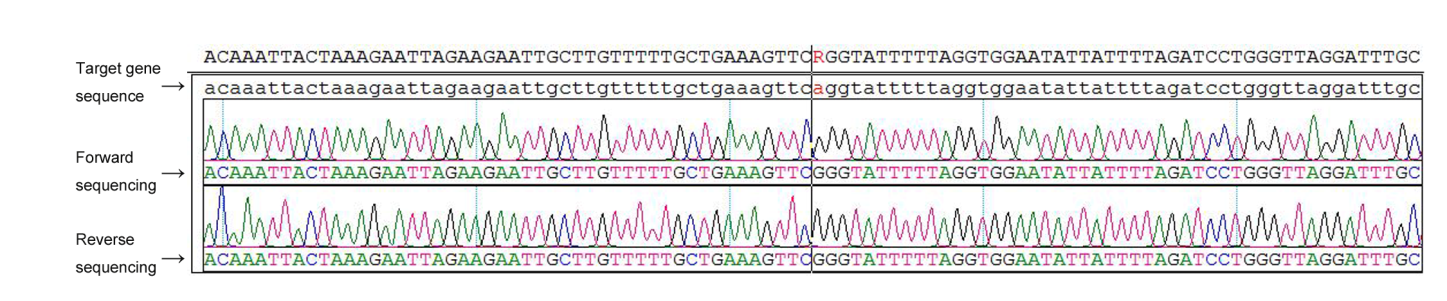


**Fig 2. Sequence diagram of DHPS PCR amplification products**


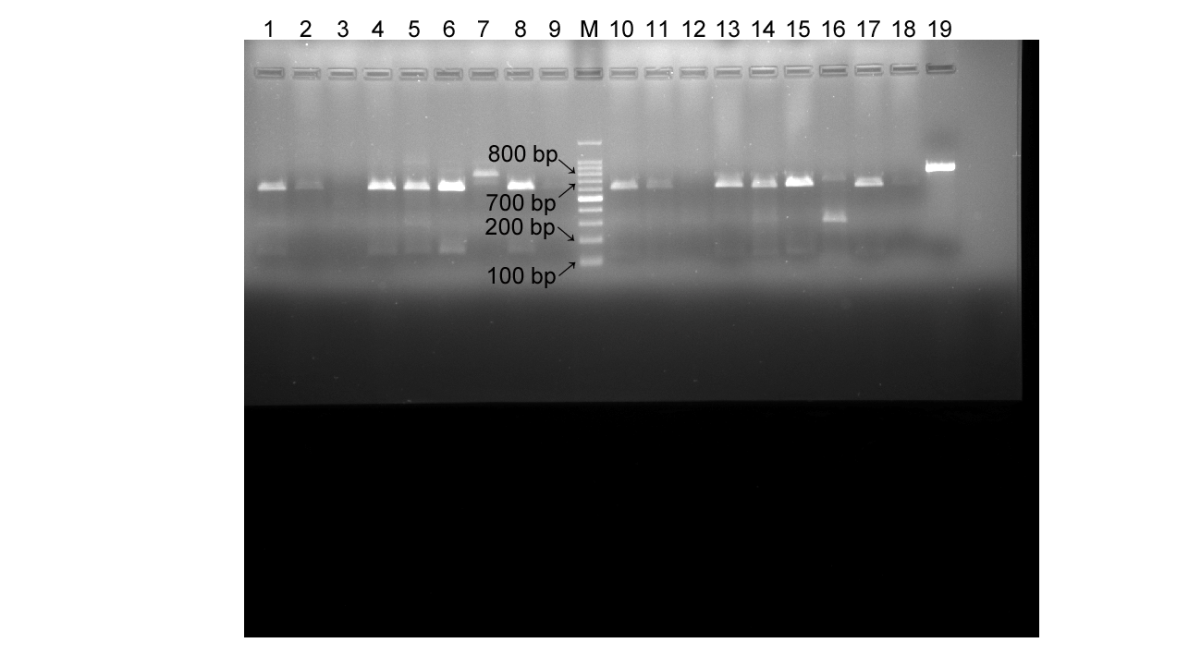


**Fig 3. A typical result of Acc I and Hae III restriction enzyme analysis of DHPS gene PCR products.** M: 100 bp DNA marker; Lanes 1–9: Results of Acc I enzyme digestion of DHPS gene amplification products; Lanes 10–18: Results of Hae III digestion of DHPS gene amplification products; Lane 19: Control (no enzyme).
